# The Clinical Utility of a Next-Generation Sequencing-Based Approach to Detecting Circulating HPV DNA in Patients with Advanced Anal Cancer

**DOI:** 10.3390/cancers17020308

**Published:** 2025-01-19

**Authors:** Deepak Bhamidipati, Jay R. Johnson, Kangyu Lin, Helene Pelicano, Cathy Eng, Ryan Huey, Robert A. Wolff, Daniel M. Halperin, Michael F. Frumovitz, Ignacio I. Wistuba, Dzifa Y. Duose, Saradhi Mallampati, Rajyalakshmi Luthra, Van K. Morris

**Affiliations:** 1 Department of Cancer Medicine Fellowship Program, The University of Texas MD Anderson Cancer Center, Houston, TX 77030, USA; deepak.bhamidipati@scri.com; 2 Sarah Cannon Research Institute, Nashville, TN 37203, USA; 3 Department of Translational and Molecular Pathology, The University of Texas MD Anderson Cancer Center, Houston, TX 77030, USA; jrjohnson5@mdanderson.org (J.R.J.); iiwistuba@mdanderson.org (I.I.W.); dyduose@mdanderson.org (D.Y.D.); rluthra@mdanderson.org (R.L.); 4 Department of Gastrointestinal Medical Oncology, The University of Texas MD Anderson Cancer Center, Houston, TX 77030, USA; klin1@mdanderson.org (K.L.); rwhuey@mdanderson.org (R.H.); rwolff@mdanderson.org (R.A.W.); dmhalperin@mdanderson.org (D.M.H.); 5 Department of Hematopathology, The University of Texas MD Anderson Cancer Center, Houston, TX 77030, USA; hpelican@mdanderson.org; 6 Division of Hematology and Oncology, Vanderbilt University Medical Center, Nashville, TN 37232, USA; cathy.eng@vumc.org; 7 Vanderbilt-Ingram Cancer Center, Nashville, TN 37232, USA; 8 Department of Gynecologic Oncology and Reproductive Medicine, The University of Texas MD Anderson Cancer Center, Houston, TX 77030, USA; mfrumovitz@mdanderson.org; 9 Department of Pathology, University of Arkansas for Medical Sciences, Little Rock, AR 72205, USA; smallampati@uams.edu

**Keywords:** anal cancer, HPV, circulating DNA

## Abstract

Background: To extend the practicality of liquid biopsy beyond the historical HPV circulating tumor DNA (ctDNA) assays, we evaluated the clinical relevance of a novel next-generation sequencing HPV ctDNA assay in patients with locally advanced and metastatic squamous cell cancer of the anal canal (mSCCA). Methods: ctDNA isolated from the plasma of patients with mSCCA was sequenced using a 1.4 Mb hybrid-capture target-enrichment panel covering the whole genome sequences of all 193 HPV types. The HPV type, copy number (CN), and integration sites were determined using a bioinformatic pipeline. Results: A total of 77 plasma samples from 28 patients with HPV-related SCCA were retrospectively analyzed. HPV ctDNA was detected in 26 cases (93%) (including uncommon subtypes). The median HPV CN was higher in metastatic versus locally recurrent/unresectable SCCA (*p* = 0.043). Changes in the HPV CN were concordant with the radiographic response (*p* = 0.027). An integration event was detected in 23 patients (82%), with presumed episomal HPV DNA present in the remaining patients. Higher HPV integration (a mean of ≥1 integration across samples) was associated with a worse overall survival from the start of immunotherapy (13.6 months versus 36.0 months; *p* = 0.003). Conclusions: Using HPV-informed next-generation sequencing of the ctDNA, we found changes in the HPV CN correlated with the treatment response and that HPV integration detected in the ctDNA is an unfavorable prognostic biomarker.

## Simple Summary

Detecting circulating tumor DNA (ctDNA) in the blood has emerged as a valuable method for evaluating several different cancers. Most anal cancers are caused by HPV infection, which is typically identified through the examination of tissue specimens. In this study, we describe a novel method for detecting HPV in the ctDNA using next-generation DNA sequencing on blood samples from patients with advanced anal cancer. The assay was able to detect multiple types of HPV, including uncommon variants. Additionally, the assay appeared to correlate with the disease burden and response to treatment. HPV DNA can often integrate into cancer DNA, which was detectable using our assay, and we show that this may prognosticate which patients are more resistant to treatment.

## 1. Introduction

The majority of new diagnoses of squamous cell carcinoma of the anus (SCCA) occur because of chronic human papilloma virus (HPV) infection [1]. Despite the availability of HPV vaccines as primary prevention of the development of HPV-associated cancers, the annual incidence of SCCA continues to rise in the United States [2]. The high-risk genotype HPV-16 is implicated most frequently in the development of SCCA. However, other high-risk types like HPV-18, -31, -33, and -45 (among others) may also promote SCCA oncogenesis [3]. Most available assays assess for the presence of HPV in anal cancer either using p16 expression through immunohistochemistry as a surrogate for the presence of HPV (agnostic to HPV type) or through DNA in situ hybridization for one or two of the most prevalent HPV types. Such analyses may often be limited by a lack of available tumor tissue for this rare malignancy.

While multimodality treatments can be curative for localized, non-metastatic SCCA, surgically unresectable or metastatic disease is associated with an estimated 5-year overall survival (OS) of less than 40%, with few effective therapeutic options in this setting [4]. Moreover, there remains an unmet need to validate biomarkers with clinical utility for the management of patients with metastatic SCCA.

The HPV genome encodes DNA sequences for six early (E) proteins which are important for viral gene regulation [5,6]. Upon the initial infection of squamous cells, the E6 and E7 HPV oncoproteins negatively regulate tumor suppressors p53 and retinoblastoma (Rb), respectively, to promote cell proliferation and, eventually in some cases, malignant transformation. Integration of the viral genome within the host genome occurs frequently and is thought to contribute to oncogenesis [7]. When it is not integrated into the genome, HPV may persist as episomes within the cytoplasm for the expression of viral proteins. Due to the presence of HPV DNA in HPV-associated cancers, the prospect of detecting HPV circulating tumor DNA (HPV ctDNA) in blood samples to monitor patients undergoing treatment is feasible. One method for detecting HPV ctDNA is droplet digital polymerase chain reaction (ddPCR), with the primers and probes targeting commonly amplified and conserved sequences such as the genes for HPV-16 and HPV-18 E7 [8]. These assays are sensitive for the detection of HPV ctDNA as a diagnostic surrogate for disease presence and for quantifying changes over time in relation to the response to treatment [9,10]. One limitation of ddPCR as a methodology is its inclusion of a limited number of HPV-specific genes and oncogenic types.

In order to improve the clinical utility of liquid biopsy for the management of HPV-associated cancers, we created a next-generation sequencing (NGS) ctDNA assay for characterizing HPV as a novel blood-based tool. In comparison to the ddPCR approaches, NGS methods have several potential advantages: the entire HPV genome is captured to increase the sensitivity, multiple HPV genotypes can be detected without prior knowledge of the genotypes involved, and integration events/sites can be identified. Comprehensive NGS-based approaches have been evaluated in HPV-associated cervical and oropharyngeal cancer, with a notably improved sensitivity compared to that of digital droplet PCR (ddPCR) [11]. However, their performance and prognostic utility in advanced anal cancer have not been explored. Herein, we report on the utility of a hybrid-capture-based NGS ctDNA assay in a prospective cohort of patients with incurable SCCA receiving systemic therapy.

## 2. Materials and Methods

### 2.1. Patients

Blood samples were prospectively obtained from patients with newly diagnosed or recurrent metastatic or surgically unresectable squamous cell carcinoma of the anal canal on a standardized IRB-approved institutional protocol at the University of Texas MD Anderson Cancer Center (MDA) and the National Cancer Institute (NCI). For each patient, 1 to 4 blood samples were collected at various time points from patients receiving systemic therapy between September 2015 and June 2019. There were two cohorts with available plasma samples for analysis with differing clinicopathologic information available: the MDA cohort (*n* = 20), in which blood was collected from patients on a variety of systemic therapies, and the extramural cohort (*n* = 8), which consisted of patients enrolled in a phase II study of the use of nivolumab (NCI9673, part A; a single-arm trial of nivolumab for patients with treatment-refractory metastatic or unresectable SCCA) in patients with metastatic anal cancer (NCT02314169). The patients in the MDA cohort received the following treatments: nivolumab alone (*n* = 5), ipilimumab and nivolumab (*n* = 4), pembrolizumab (*n* = 2), atezolizumab plus bevacizumab (*n* = 2) (NCT03074513), or durvalumab plus the HPV-16/18 E6/E7 therapeutic DNA vaccine MEDI-0457 (*n* = 1) (NCT03439085). Clinical variables such as prior treatments, treatment response, and follow-up were collected from their electronic medical records. Radiographic increases and decreases in tumor burden were determined by the treating physician and through multidisciplinary review; the RECIST v1.1 criteria were instituted for the NCI cohort. The full treatment history and baseline characteristics were available for the MDA cohort; meanwhile, limited information, including age, metastatic sites, date of progression on trial treatment, and date of last follow-up, were available for the NCI cohort. All of the participants signed informed consent according to an IRB-approved protocol prior to the conduction of any experimental research studies using specimens that they provided optionally.

### 2.2. Sample Processing and the Bioinformatic Pipeline

Frozen plasma samples were thawed in a room-temperature water bath and centrifuged at 1600× *g* for 10 min to remove precipitated debris. CtDNA was isolated from the supernatant using the QIAsymphony DSP Circulating DNA Kit (Qiagen, Germantown, MD, USA) on the QIAsymphony automated instrument per the manufacturer’s instructions. Libraries were prepared with input DNA of 5 ng to 30 ng using the NEBNext Ultra II DNA Library Prep Kit (New England Biolabs, Ipswich, MA, USA) with the addition of dual molecular barcode adaptors synthesized from single-stranded sequences obtained from IDT (IDT Technologies, Coralville, IA, USA). Briefly, the ctDNA underwent end-repair and adaptor ligation before purification using SPRIselect beads to obtain the adaptor-ligated libraries. These libraries were amplified and then enriched for the target regions using the in-house designed HPV panel through hybridization capture. The panel has 34,048 baits and covers a 1.432 Mb genome from 193 HPV strains. The panel also includes the human TP53 gene as an internal control. Following post-hybridization capture washes, the samples were amplified and then purified using SPRIselect beads and eluted in low-TE buffer. The final libraries were quantified using the 4200 TapeStation system (Agilent Technologies, Santa Clara, CA, USA), pooled, and sequenced using the Nextseq550 per Illumina’s guidelines (Illumina, San Diego, CA, USA). A custom bioinformatics pipeline was developed to analyze the resulting sequencing data to report the HPV type, the number of HPV copies, and the integration site into the human genome. Briefly, raw sequencing reads from FASTQ files were collapsed using dual unique molecular indexes, and single- and double-consensus reads were derived. Then, these error-corrected reads were aligned with the HPV and human genomes using both the BWA and Bowtie2 tools. The resulting consensus files were then run through an in-house application to determine the involved HPV type and the fraction of the HPV genome covered in each sample. The number of HPV copies present in each sample was determined using a calculation based on the read coverage of each HPV type. GRIDSS was used to determine the viral integration site for each HPV type.

### 2.3. Statistical Analysis

Demographic and clinical variables were summarized using descriptive statistics, with frequency (%) for categorical variables and the median for continuous variables. Comparisons between continuous variables were conducted using the Mann–Whitney *U* test, while comparisons between categorical groups were conducted using the nonparametric Fisher’s exact test when applicable. The Kaplan–Meier method was used to estimate the median survival. Progression-free survival (PFS) was defined as the time from treatment initiation to the time until disease progression or patient death. Overall survival (OS) was defined as the length of time from a diagnosis or treatment initiation until a patient’s death from any cause. Cox’s proportional hazard regression models were applied to assess the association between the patients’ characteristics and the time to event outcomes while controlling for covariates. Our study was intended to be descriptive in nature, as opposed to hypothesis-testing, and *p* values are presented without correction. For all analyses, a *p* value of ≤0.05 was considered statistically significant. All of the statistical analyses were conducted using SPSS version 29.

## 3. Results

### 3.1. Patient Characteristics

A total of 28 patients underwent prospective blood collection for the detection of the HPV copies and integration sites in the ctDNA at multiple time points for a total of 77 analyzed samples (range of 1–4 samples per patient). Their baseline characteristics are summarized in Table 1. Their median age was 60 years, and most patients had received treatment for locoregional disease before the metachronous development of advanced/unresectable disease. In total, 22 patients (71% of patients) received an anti-PD-1 immune checkpoint inhibitor, of whom most patients received nivolumab alone (*n* = 13) or in combination with ipilimumab for metastatic disease (*n* = 4, Supplementary Table S1).

### 3.2. HPV Copy Number Changes with Treatment

**Figure 1:**
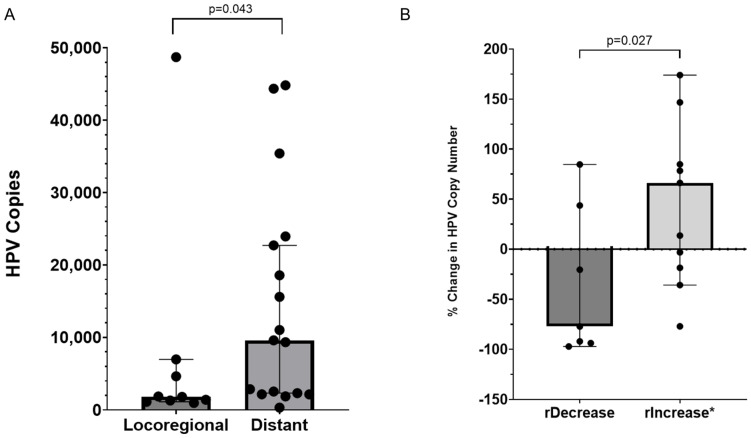
HPV copy number and clinical endpoints. (**A**) displays the highest HPV copy number detected among patients with locoregional and distant metastatic disease (the bar represents the median and each dot represents a patient). (**B**) Percentage change in HPV copy number for paired samples according to radiographic response. * One value with a 1692% increase is not depicted. rDecrease = radiographic decrease and rIncrease = radiographic increase.

Among the patients with detectable HPV (*n* = 26), the maximum HPV copy number at any time point was higher among patients with distant metastatic disease relative to those with locally advanced/surgically unresectable disease. The median copies of HPV in locoregional disease (*n* = 9) were 1805 [Standard Deviation (SD): 15,530] versus 9462 (SD: 15,243) among patients with distant metastatic disease (*n* = 17) (*p* = 0.043) (Figure 1A).

A total of 15 patients had more than one collection time point, for whom there were serial on-treatment blood collections with associated radiographic outcome information available for determining the correlation between their ctDNA HPV copy number changes and radiographic response. In total, there were 18 time points with paired samples at least 28 days apart for evaluating dynamic changes in ctDNA in response to treatment. The median time between blood collections was 82 days (range: 28–274 days).

Changes in the HPV ctDNA burden appeared to correlate with the response to treatment for incurable SCCA. A radiographic decrease in the disease burden was observed between the collections for 7 paired samples, and a radiographic increase in the disease burden was observed between the collections for 11 paired samples. The percentage change in the HPV copy number for paired samples according to the radiographic response to treatment is displayed in Figure 1B. Among patients with a radiographic decrease in disease on treatment, the median percentage decrease in their HPV copy number was 77.3% versus a median increase of 66.2% among the patients with a radiographic increase (*p* = 0.027).

### 3.3. HPV Variants and the Detection of HPV’s Integration into the ctDNA

The detected HPV variants and a description of the integration events were available for all patients (the MDA and NCI cohorts). Beyond HPV-16, additional oncogenic HPV variants were detected in the plasma samples: HPV-18 (*n* = 2), HPV-45 (*n* = 2), HPV-73 (*n* = 1), and HPV-91 (*n* = 1) were detectable in the blood of five patients (Table 2). HPV-18, HPV-45, HPV-73, and HPV-91 were concurrent with HPV-16 in four patients, and HPV-18 and HPV-45 were concurrent in one patient (Supplementary Figure S1). A total of 298 integration events were detected in 70 of the plasma samples analyzed. A median of two integration events (range: 0–46) was detected per sample. There were 26 samples with detectable HPV and no concomitant integration events, which we attributed to a non-integration, episomal HPV-associated event.

**Figure 2:**
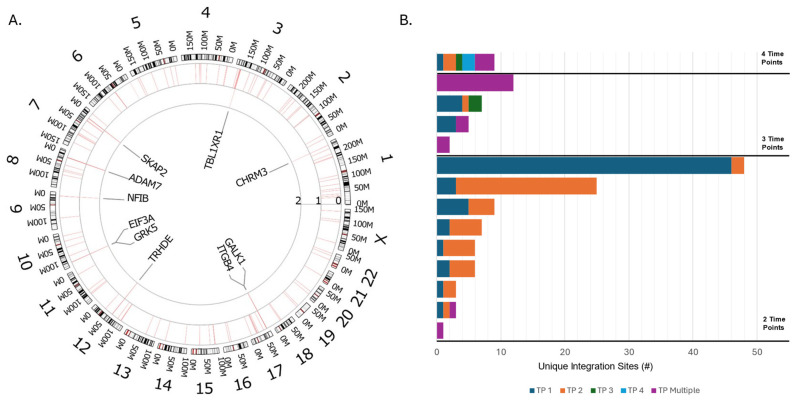
Integration hotspots and concordance. (**A**) The distribution of HPV integration breakpoints in the human genome among all patients with detectable HPV (*n* = 26). In the outer circle, each chromosome is represented, with the DNA numbering in millions of bases. In the inner circle, each red bar depicts the frequency of HPV integration, where the histogram axis units represent the number of samples. The tumor-associated genes located <500 kb from high rates of integration breakpoints are marked. (**B**) The number of unique integration events detected across multiple blood samples for patients with ≥2 samples. Each bar represents an individual patient, and the x-axis refers to the number of distinct integration loci. The color code denotes whether the integration loci were detected at an individual time point or at multiple time points. TP = time point.

We identified 127 HPV integration breakpoints involving 27 genes across the analyzed genome (Table 3). There were eight integration “hotspots” with a breakpoint frequency of ≥2 within 500 kbs of the following genes (Figure 2A): *ADAM7*, *SKAP2*, *TBL1XR1*, *CHRM3*, *GALK1*, *ITGB4*, *THRDE*, *GRK5*, *EIF3A*, and *NFIB*.

In most cases, unique HPV integration events were detected across serial samples collected from the same patient at multiple timepoints. However, among 14 patients with integration events detected in more than one sample, 6 patients had at least one integration site detected at multiple time points (Figure 2B).

### 3.4. HPV’s Integration into ctDNA as a Prognostic Marker

**Figure 3:**
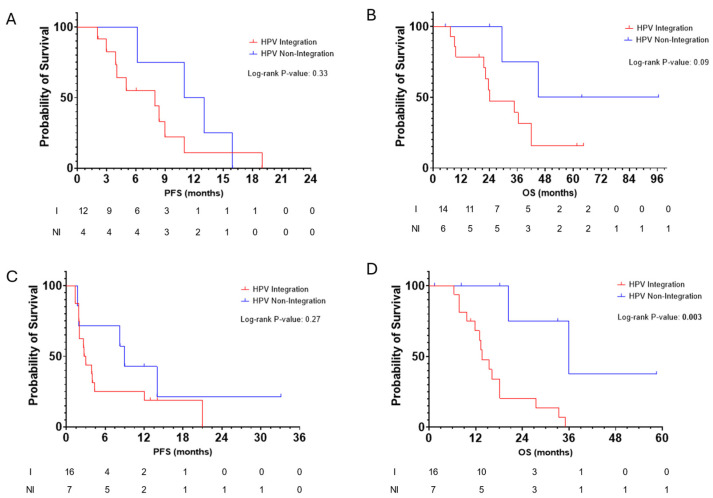
Survival according to HPV integration status. Survival according to HPV integration status: The PFS (**A**) and OS (**B**) according to all systemic treatments; PFS (**C**) and OS (**D**) after the initiation of immunotherapy. I = integration group; NI = non-integration group.

Patients with detectable HPV ctDNA were classified as “HPV integration” or “HPV non-integration” cases according to the detection of integration events in order to evaluate HPV integration status as a prognostic biomarker following systemic treatment for incurable SCCA. Patients were designated as “HPV non-integration” cases if no integration events were detected at any time point or the mean number of integration events across multiple samples was less than 1 (*n* = 9). All of the other patients were designated as “HPV integration” cases (*n* = 19). We first evaluated the survival outcomes for patients with incurable SCCA after all types of systemic treatment (e.g., cytotoxic chemotherapy, immunotherapy, and/or targeted therapies like use of the anti-EGFR antibody cetuximab). In the MDA cohort with its full treatment history available (*n* = 20), the median PFS with first-line chemotherapy for metastatic disease was 8.0 months (95% CI: 0 months to 16 months) versus 11 months (95% CI: 4.2 months vs. 17.7 months) in the HPV integration group versus the HPV non-integration group (HR: 0.59; 95% CI:0.20 to 1.72; *p* = 0.33) (Figure 3A). The median OS from a diagnosis of metastatic disease was 24.3 months (95% CI: 5.3 months to 43.3 months) versus not reached in the HPV integration group versus the HPV non-integration group (HR: 0.30; 95% CI: 0.10 to 0.92; *p* = 0.09) (Figure 3B).

We next evaluated the outcomes according to HPV integration based on treatment with immunotherapy among the patients with available data (from the MDA cohort and the NCI cohort; *n =* 23). In this cohort, the median PFS from the start of PD(L)-1 inhibitor use for metastatic disease was 2.8 months (95% CI: 2.1–3.5 months) versus 9.0 months (95% CI: 7.2–10.9 months) in the HPV integration group versus the HPV non-integration group (HR: 0.58; 95% CI: 0.23–1.45; *p* = 0.27) (Figure 3C). The median OS from the initiation of a PD(L)-1 inhibitor was 36.0 months (95% CI: 10.5 months–16.8 months) versus 13.6 months (95% CI: 12.8–59.1 months) (HR: 0.21; 95% CI: 0.06–0.42; *p* = 0.003) (Figure 3D) in favor of the non-integration event. In a multivariable model including age and the extent of disease (locoregional only versus distant) at the start of anti-PD(L)1 therapy, the association between integration status and OS from the start of immunotherapy remained significant (HR: 0.17; 95% CI: 0.03–0.90; *p* = 0.037).

## 4. Discussion

Non-invasive approaches such as analysis of the ctDNA provide valuable information for guiding the treatment and monitoring decisions for patients with rare malignancies like SCCA, for which tumor tissue is often not available. In this study, we report that analysis of the circulating HPV DNA in the peripheral blood using an HPV-informed next-generation sequencing approach correlated not only with the disease burden but also with the response to systemic treatment for patients with incurable SCCA. Here, we extend the applicability of liquid biopsy beyond HPV ddPCR for anal cancer by characterizing genomic events using a tissue-agnostic methodology. For example, integration events were detectable in the ctDNA and were prognostic with respect to the overall survival after the initiation of immunotherapy. Our work adds to the growing body of literature for other HPV-associated cancers which suggests that ctDNA analysis, such as whole HPV genome sequencing of the circulating HPV DNA, represents a feasible approach to monitoring patients receiving treatment for HPV-positive anal cancer [12].

Initial studies utilizing ctDNA in advanced anal cancer primarily examined ddPCR assays in clinical cohorts, which detect 100–200 bp sequences in the HPV genome of the most common strains [13]. Compared to ddPCR, broad NGS of the HPV DNA theoretically has improved sensitivity due to its ability to detect less frequent genotypes; though this was limited by the small sample size in this pilot study, all of the patients had detectable HPV ctDNA in the present study. Highlighting this concept, a study which compared ddPCR and NGS for the detection of HPV copies in patients with HPV-positive cervical and oropharyngeal cancer found a strong correlation between the HPV copies detected by each approach; however, the NGS-based assay was able to detect lower HPV copy numbers than ddPCR—a finding which has also been demonstrated in a meta-analysis [11,14]. One such NGS panel which was evaluated in patients with localized anal cancer comprised two primer pools covering distinct regions of eight high-risk HPV genomes [15]. Comparatively, our panel, designed to detect all HPV genotypes, was able to detect multiple high- and low-risk HPV genotypes over multiple time points. Moreover, our assay was able to detect integration events in the ctDNA, which had the potential to provide prognostic information.

Currently, there are no established blood-based surrogates for quantifying the tumor burden and treatment response beyond radiographic imaging; thus, circulating markers such as HPV DNA may have an important role in patients with advanced anal cancer. As in our study, an ancillary analysis of the Epitopes-HPV02 trial assessing frontline docetaxel, cisplatin, and 5-FU (DCF) for advanced/unresectable anal cancer identified a relationship between the extent of disease (localized versus distant metastasis) and HPV ctDNA copies using ddPCR, as well as dynamic changes, in the setting of systemic therapy [10]. More commonly, HPV ctDNA has been evaluated as a predictive biomarker for early recurrence after the administration of definitive chemoradiotherapy for localized disease [9,15,16]. In a study which included paired pre- and post-treatment samples from patients receiving definitive chemoradiotherapy for anal cancer, HPV ctDNA was detectable in three patients, and all of these patients developed relapse within 6 months [9]. In the advanced setting, circulating HPV ctDNA has several potential uses: serial monitoring of the response to therapy prior to conventional imaging may provide an opportunity to prompt early intervention such as imaging, and these assays may also be used as a sensitive method for detecting residual disease, such as in cases of patients who have a durable response to immunotherapy but with radiographic findings equivocal to those in residual disease.

There appeared to be an association between HPV DNA integration and the long-term outcomes after receiving systemic therapies such as immunotherapy in the present study. In the Epitopes-HPV2 trial, HPV integration was associated with a trend towards a worse PFS on DCF, with a median PFS of 10.6 months vs. 19.5 months, though this was not statistically significant [17]. Several studies have suggested the possible immunomodulatory effect of HPV’s integration into the cancer genome. In a Cancer Genome Atlas (TCGA) cohort of HPV-positive head and neck cancers (HNSCCs) analyzed using RNA-sequencing to detect viral–host fusion transcripts as a marker of integration, HPV-positive integration-negative cases were associated with a superior OS compared to that of HPV-positive, integration-positive cases [18]. The RNA sequencing of HNSCC (*n* = 84) revealed that non-integrated cases had stronger immune signatures, characterized by heightened signatures for CD4+ T-cells, CD8+ T-cells, CD3+ T-cells, NK cells, regulatory T-cells, B cells, NK T-cells, and CD34+ cells. In other studies analyzing samples from patients with HNSCC, *MYC* and *PDL1* were identified to be common integration hotspots, and integration was related to the increased expression of these genes [19,20]. Similarly, in cervical adenocarcinomas, the integration of HPV DNA into hotspots such as STARD3 and ERBB2 was correlated with increased protein expression pf STARD3 and ERBB2, respectively [21]. Possibly due to the small sample size and differing underlying biology, the hotspots identified in the present study did not overlap with the previously identified hotspots in HPV-positive HNSCC and cervical cancer [20,22]; however, several of these hotspot genes have been associated with tumor progression and metastasis [23,24,25,26,27,28,29,30]. Taken together, it appears that HPV integration alters cancer gene transcription to promote treatment resistance, but it is unclear how the integration of the HPV genome into these various hotspots influences tumorigenesis, and further work is needed to elucidate this.

There were several limitations to the present study. First, the sample size was limited, and the full clinical history was not available for a significant number of the patients, so the survival outcomes from the time of diagnosis and the lines of therapy were unable to be analyzed for all patients. Therefore, it is unclear whether survival from the initiation of immunotherapy represents a true prognostic benefit of non-integration status or a reflection of heterogenous post-progression therapy. Notably, there are no standard-of-care therapies approved after progression on immunotherapy. The blood collection time points were also heterogeneous within and between patients, so this study was unable to establish a quantitative biomarker based on a consistent time point and treatment regimen. There were also several cases where the HPV ctDNA count was discordant with the radiographic response, which highlighted the inherent variability in the ctDNA, which may have been related to a variety of preanalytical and patient factors.

## 5. Conclusions

In conclusion, we demonstrate the clinical applicability of a whole HPV genome sequencing ctDNA assay in a cohort of patients with advanced anal cancer with respect to the treatment response and establish the potential prognostic role of HPV DNA integration detected in the ctDNA. These data suggest that further prospective validation of this assay is warranted, including the prognostic role of the integration detected in the ctDNA.

## Figures and Tables

**Table 1:** Baseline characteristics.

Characteristic	MDA Cohort (%), *n* = 20	NCI Cohort (%), *n =* 8
Median age (range, y)	60 (44–72)	61 (36–70)
Gender		-
Male	4 (20%)	
Female	16 (80%)	
Initial staging at diagnosis		-
Locoregional	14 (70%)	
Metastatic	6 (30%)	
HPV status		-
p16+ according to immunohistochemistry	20 (100%)	
Treatments for advanced disease		-
5-fluorouracil and cisplatin	9 (45%)	
Carboplatin and Paclitaxel	9 (45%)	
Immunotherapy	12 (60%)	
Cetuximab	4 (20%)	
Site of recurrence or metastases		
Locoregional only	7 (35%)	3 (38%)
Distant	13 (65%)	5 (63%)
Median follow-up from metastatic disease (months)	26.9	-

**Table 2:** HPV types detected from ctDNA.

HPV Type	# of Patients (*n* = 28)
HPV-16	25
HPV-18	2
HPV-45	2
HPV-73	1
HPV-91	1

**Table 3:** Protein-coding genes encompassing HPV integration breakpoints.

Gene Symbol	Chromosome	Description
CHRM3	1	cholinergic receptor, muscarinic 3
FMN2	1	formin 2
GREM2	1	gremlin 2, DAN-family BMP antagonist
TBL1XR1	3	transducin (beta)-like 1 X-linked receptor 1
SKAP2	7	src kinase-associated phosphoprotein 2
ADAM7	8	ADAM metallopeptidase domain 7
ADAMDEC1	8	ADAM-like decysin 1
NFIB	9	nuclear factor I/B
EIF3A	10	eukaryotic translation initiation factor 3, subunit A
FAM45A	10	family with sequence similarity 45, member A
GRK5	10	G protein-coupled receptor kinase 5
NANOS1	10	nanos homolog 1 (Drosophila)
PRDX3	10	peroxiredoxin 3
SFXN4	10	sideroflexin 4
CASKIN2	17	CASK-interacting protein 2
GALK1	17	galactokinase 1
ITGB4	17	integrin, beta 4
KIAA0195	17	KIAA0195
LLGL2	17	lethal giant larvae homolog 2 (Drosophila)
MYO15B	17	myosin XVB
RECQL5	17	RecQ protein-like 5
SAP30BP	17	SAP300binding protein
SMIM5	17	small integral membrane protein 5
SMIM6	17	small integral membrane protein 6
TSEN54	17	TSEN54 tRNA splicing endonuclease subunit

## Data Availability

The data generated in this study are available upon request from the corresponding author.

## Supplementary Materials

The following supporting information can be downloaded at https://www.mdpi.com/article/10.3390/cancers17020308/s1. Table S1. Immunotherapy regimens; Figure S1. HPV Subtypes.
