# Supplementary material for: The Clinical Utility of a Next-Generation Sequencing-Based Approach to Detecting Circulating HPV DNA in Patients with Advanced Anal Cancer
Source: Cancers (Basel). 2025 Jan 19;17(2):308. doi: 10.3390/cancers17020308 (PMC11764299; doi:10.3390/cancers17020308)
Supplement: Supplementary file 1 [file cancers-17-00308-s001.zip › cancers-3383903-supplementary.pptx]

## Slide 1
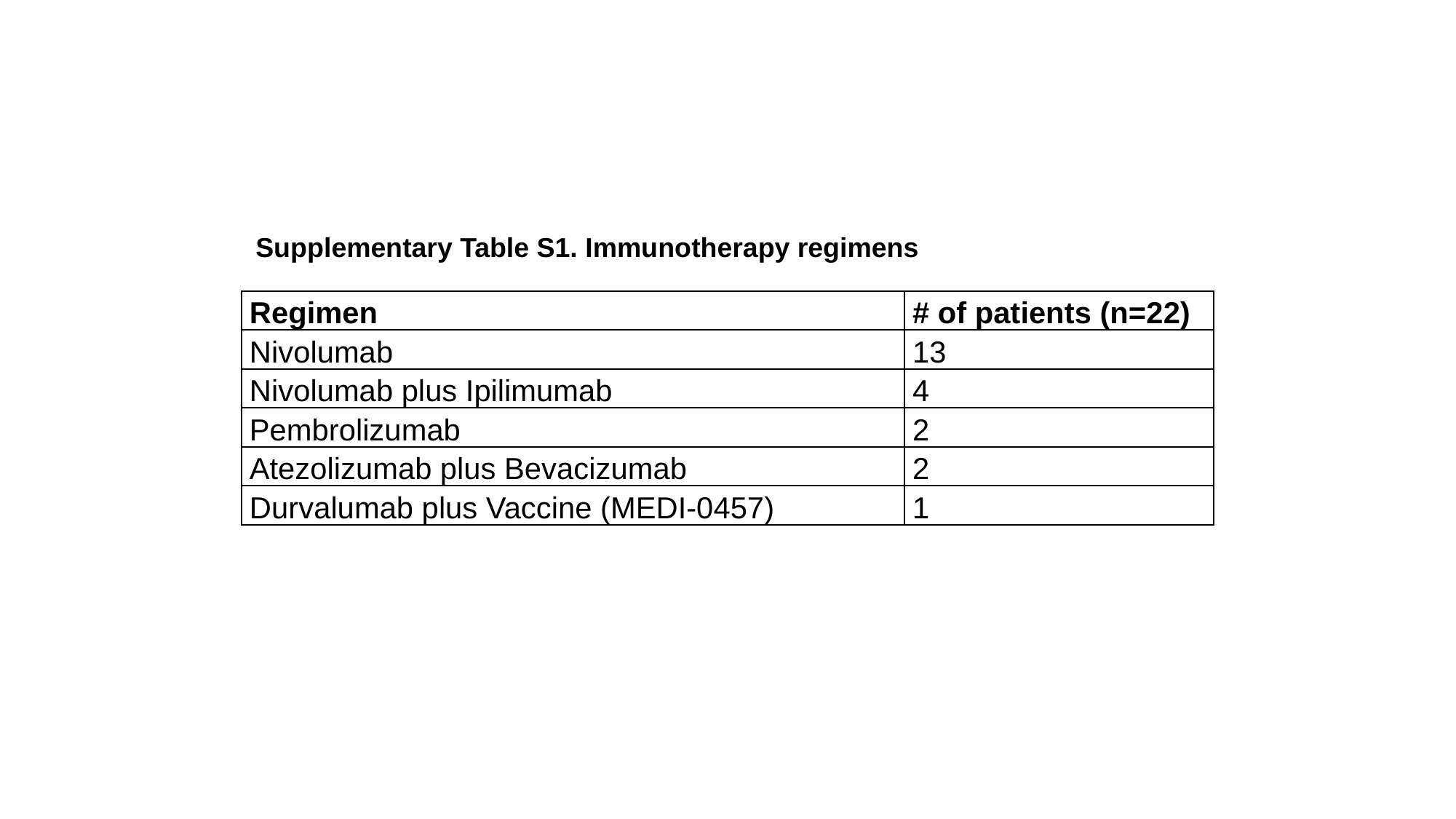

Supplementary Table S1. Immunotherapy regimens
| Regimen | # of patients (n=22) |
| --- | --- |
| Nivolumab | 13 |
| Nivolumab plus Ipilimumab | 4 |
| Pembrolizumab | 2 |
| Atezolizumab plus Bevacizumab | 2 |
| Durvalumab plus Vaccine (MEDI-0457) | 1 |

## Slide 2
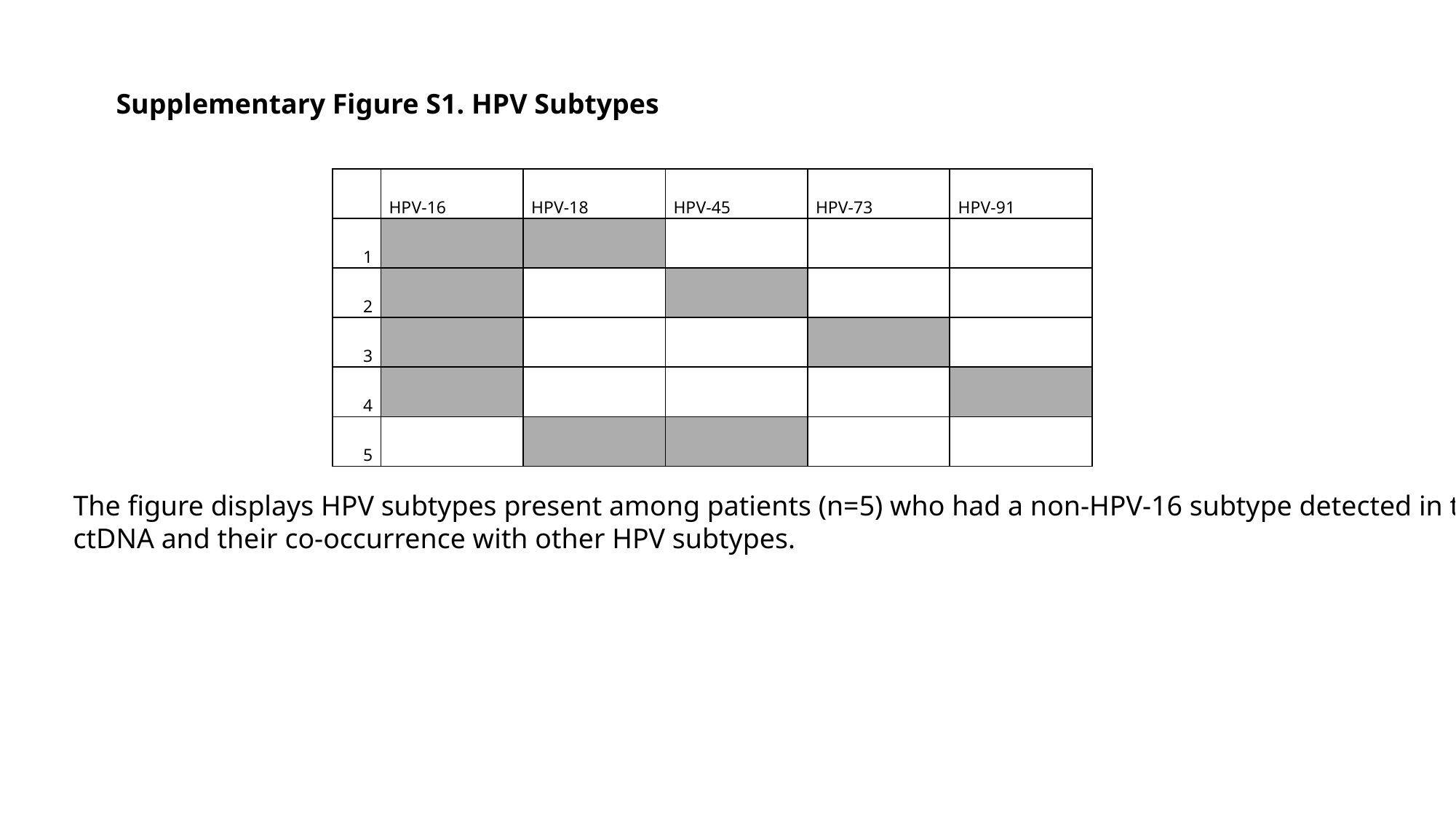

Supplementary Figure S1. HPV Subtypes
| | HPV-16 | HPV-18 | HPV-45 | HPV-73 | HPV-91 |
| --- | --- | --- | --- | --- | --- |
| 1 | | | | | |
| 2 | | | | | |
| 3 | | | | | |
| 4 | | | | | |
| 5 | | | | | |
The figure displays HPV subtypes present among patients (n=5) who had a non-HPV-16 subtype detected in the
ctDNA and their co-occurrence with other HPV subtypes.
